# Analysis of correlation between the consumption of beverages and the risk of radiographic knee osteoarthritis in Korean people: A cross-sectional study using the Fifth Korea National Health and Nutrition Examination Survey (KNHANES V-1, 2)

**DOI:** 10.1097/MD.0000000000030265

**Published:** 2022-09-16

**Authors:** Chae Ouk Lim, Hyung Jun Park, Bong Mo Koo, Bo Taek Kim, Jae Gyoon Kim, Gi Won Choi

**Affiliations:** a Department of Orthopedic Surgery, Korea University College of Medicine, Ansan Hospital, Gyeoung-gi-do, South Korea; b Department of Orthopedic Surgery, Korea University College of Medicine, Ansan Hospital.

**Keywords:** coffee, knee, osteoarthritis, polyphenol, women

## Abstract

The purposes were to analyze correlations between the frequency of beverage drinking (coffee, green tea, milk, and soft drinks) and the presence of radiographic knee osteoarthritis (OA) in relation to sex. We performed this study using the Korea National Health and Nutrition Examination Survey (KHANES V-1, 2). We examined data from 5503 subjects after exclusion. We utilized the food frequency questionnaires from KHANES, and reorganized them into 2 or 3 groups according to the frequency of beverage consumption. We analyzed the relationship between radiographic knee OA and beverage consumption statistically after adjusting confounding factors with multivariable logistic regression analysis. Knee OA was inversely associated with coffee consumption only in women (*P* < .05). The odds ratio of knee OA was lower in those who drank at least a cup of coffee than in those who did not drink coffee in women (*P* for trend < .05). However, there was no significant linear trend of the odds ratio of each group in both sexes for drinking other beverages. As the coffee consumption increased, the radiographic knee OA group showed decreasing linear trend only in women. However, other beverages did not show a significant relation to the radiographic knee OA in both sexes.

## 1. Introduction

Osteoarthritis (OA) is a multifactorial, age-dependent, degenerative disease characterized by articular cartilage loss, osteophyte formation, and subchondral bony sclerosis.^[1]^ The only fundamental treatment for patients with end-stage OA is joint replacement arthroplasty, which accounts for a huge healthcare burden.^[2,3]^ An in-depth understanding of the pathogenesis and risk of OA is helpful for its early prevention.^[4]^ However, the risk factors for OA are not well known, and it is important to find modifiable risk factors for OA progression.^[5]^ A major, potentially modifiable lifestyle factor is diet. Previous research with respect to dietary risk factors for OA has focused mainly on overall nutritional intake such as body mass index (BMI), rather than on specific foods.^[6,7]^ In addition, no population-based studies have examined the relationship between common beverage consumption and knee OA in Korea or worldwide.

Coffee is one of the most common beverages taken worldwide. South Korea has undergone large shifts in diet over the past decade and has experienced food consumption style changes due to rapid economic growth and the introduction of western food culture.^[8]^ Coffee consumption has increased over the past decades with shifts in diet.^[9]^ Coffee is not only the primary source of caffeine but also a major source of antioxidants in the diet.^[10]^ Tea has been also consumed worldwide, and green tea is also one of the favorite beverages in Korea. Green tea contains polyphenolic flavonoids, which have been known as antioxidant and anti-inflammatory materials.^[11]^ Milk is an excellent source of vitamins and minerals, dairy calcium, and protein. It has long been recognized for its important role in bone health.^[12]^ A few studies indicated that milk consumption reduces knee OA progression partially through elevated dietary calcium intake.^[13,14]^ Soft drinks are non-alcoholic, carbonated, sugar-sweetened beverages. Soft drink has been consumed widely across the globe in recent decades.^[15]^ Sugar-sweetened soft drink intake can lead to weight gain and has been associated with the risk of obesity and type 2 diabetes mellitus.^[16]^ In addition, the prevalence of knee OA differed by sex.^[17]^ Also, 1 study reported that the frequent consumption of soft drinks may be associated with increased OA progression only in men.^[5]^ However, there was only a few studies have reported the co-relationship between the consumption of beverages and the prevalence of knee OA according to sex.

The purposes of this study were to compare the frequency of beverage drinking (coffee, green tea, milk, and soft drinks) according to the presence of radiographic knee OA and to analyze correlations between the frequency of beverage drinking and the presence of radiographic knee OA in relation to sex.

## 2. Materials and Methods

### 2.1. Study overview

We used data collected during the Fifth Korea National Health and Nutrition Examination Survey (KNHANES V-1, 2) by the Korean Centers for Disease Control and Prevention for this cross-sectional study. KHANES selects the subjects using a stratified, multistage probability sampling strategy every year. As this survey has selected the sample group each year (10,000/50,000K), the same individual selected more than once would have a very low chance. Informed consent was taken from every participant in the survey. Institutional Review Board permission was not needed because academic usage of the data was already granted to the public. A self-administered questionnaire was applied to most of the survey questions. The details of the data-collecting/physical examination method used by KHANES were previously described.^[18]^

**Figure 1.**
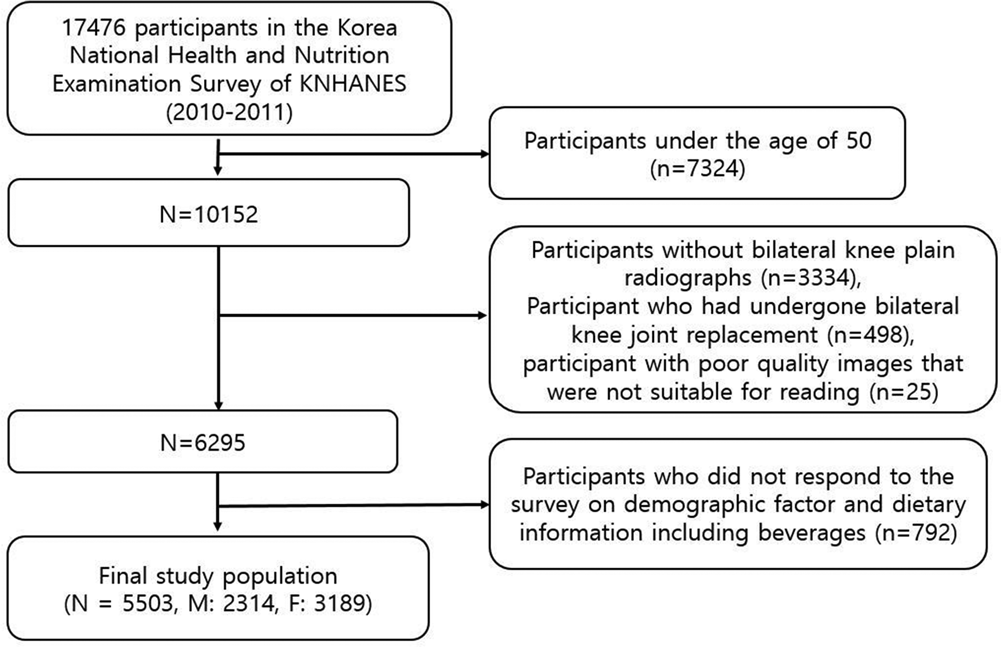
The subjects eligible for study: Fifth Korea National Health and Nutrition Examination Survey (KNHANES V-1, 2). F = female, M = male, N = number of participants.

We performed this study using research data from KNHANES V-1 (8958 people in 3840 households) in 2010 and KNHANES V-2 (8518 people in 3840 households) in 2011. Out of 17,476 participants, we included participants aged 50 years and over (N = 10,152). Then, we excluded participants with bilateral knee radiographs (N = 3334) and with bilateral knee joint replacement (N = 498), along with poor quality radiographs not suitable for reading (N = 25). Of the remaining patients, we also excluded participants who did not respond to surveys on height, body weight, waist circumference (WC), demographic factors (diabetes mellitus and hypertension history, cigarette and alcohol intake), and dietary information, including beverages, which was assessed via a 165-item food frequency questionnaire (N = 792). Overall, we included data of 5503 (2314 men and 3189 women) participants (Fig. 1).

### 2.2. Demographic and life style data

A 4-part questionnaire that included basic demographics such as age, sex, and personal medical history including diabetes mellitus and hypertension along with lifestyle habits such as alcohol drinking, smoking, and regular physical activity was completed. Participants were divided into groups in alcohol drinking over the past year (none: less than once per month, mild to moderate: greater than once per month but less than twice per week, and heavy: greater than twice per week), smoking (never smokers, past smokers, and current smokers), and regular physical activity (regular exercise: for at least 20 minutes at a time at least 3 times a week).^[18]^ BMI was defined as body weight/height^2^ (kg/m^2^) measured using standard protocols. WC was measured at the midpoint between the bottom of the rib cage and the top of the lateral border of the iliac crest with full expiration.^[19]^

### 2.3. Assessment of beverage consumption

To assess beverage consumption, we used the food frequency questionnaires from KHANES. KHANES provides 9 response choices reflecting a wide range of beverage consumption, from “almost none” to “3 cups a day.” The other options are as follows: 6–11 cups a year, 1 cup a month, 2–3 cups a month, 1 cup a week, 2–3 cups a week, 4–6 cups a week, 1 cup a day, and 2 cups a day (Table 1).

**Table 1:** The self-administered questionnaire including 10 drinking frequency categories presented to the participants.

※ How often do you have this beverage? (as “1 cup”)
Please check the appropriate number.
0. Never or hardly
1. 6–11 times a year
2. Once a month
3. 2–3 times a month
4. Once a week
5. 2–3 times a week
6. 4–6 times a week
7. Once a day
8. 2 a day
9. 3 more times a day

The standard serving size for beverages was assigned on the questionnaire as “1 cup.” In order to evaluate the effect of beverage consumption, we re-categorized the 9 responses in 2 different ways. First, we simply reorganized the 9 categories into 2 groups according to whether they were drinking or not. The reclassification is as follows: group 1 (category 0) and group 2 (categories 1–9). Secondly, the 9 categories were reclassified into 3 categories as follows: Group 1 (category 0), Group 2 (categories 1–6), and Group 3 (categories 7–9). We named group 1 as “never or hardly,” group 2 as “monthly to below daily,” and group 3 as “daily”.

### 2.4. The definition of knee OA

Bilateral weight-bearing anteroposterior and lateral (30° flexion) plain radiographs of the knee were taken using the SD 3000 Synchro Stand (Accele Ray, Shinyoung Co., Seoul, South Korea). OA degrees were assessed with the Kellgren–Lawrence grading system (KL grade).^[20]^ The radiographs were graded by 2 radiologists, and they determined grades in agreement. When there was disagreement between the 2 radiologists, the higher grade was accepted. If the grade difference was over 1 grade, the grade of the third radiologist was accepted. The higher grade was determined as the KL grade of each participant between both limbs. Inter-rater agreement within 1 grade between the 2 radiologists was 92.8%, and the weighted Cohen’s kappa coefficient was 0.65.^[19]^ Radiographic knee OA was defined as those who had a KL grade ≥2 in at least 1 knee.^[19]^

### 2.5. Statistical analyses

Demographic data of subjects according to the presence or absence of knee OA were compared by independent *t* test for continuous variables and chi-square tests for categorical variables. The chi-square test performed a comparison of the frequency of beverage consumption and the presence of radiographic knee OA. Lastly, a multivariable logistic regression analysis was conducted to evaluate the correlation of beverage drinking frequency with the existence of knee OA. Using multivariable logistic regression, we evaluated the odds ratio (OR) in relation to sex after adjusting for potential confounders: age, BMI, WC, smoking, alcohol drinking, regular physical exercise, diabetes mellitus, hypertension prevalence, and income. Then, we evaluated the OR for the existence of knee OA in each beverage consumption subgroup, referencing the lowest consumption subgroup. All analyses were performed with SPSS, version 18.0 (IBM Corp., Armonk, NY, USA). *P* values of <.05 were considered statistically significant. All analyses were performed separately by sex.

## 3. Results

### 3.1. Demographic data of subjects according to the existence of knee osteoarthritis

The prevalence of OA was 41.3% (2273/5503) in the final study participants, with a prevalence of 31.8% (736/2314) for men and 48.2% (1537/3189) for women. Patients with radiographic knee OA showed significantly higher mean age, BMI, WC, and hypertension prevalence than those without OA in both men and women (*P* < .001). The prevalence of diabetes was significantly higher for subjects with radiographic knee OA than for those without OA only in women (*P* < .001), but men did not show a significant difference. Smoking did not show significance in either sex. Alcohol consumption was significantly higher for patients with radiographic knee OA than for those without OA in both men and women (men: *P* = .003, women: *P* < .001). There was no difference in regular exercise between patients with radiographic knee OA and non-OA in either sex (Table 2).

**Table 2:** Baseline characteristics of study participants with or without radiographic knee osteoarthritis.*

	Men (N = 2314)					Women (N = 3189)		
	Non-OA	OA			*P* value	Non-OA	OA	*P* value
Numbers (%)	1578(68.2%)		736(31.8%)			1652 (51.8%)	1537 (48.2%)	
Age (yr)	59.9 ± 0.2		66.1 ± 0.4	<.001		59.3 ± 0.2	68.0 ± 0.3	<.001
Waist circumference (cm)	84.9 ± 0.2		86.5 ± 0.4	<.001		80.4 ± 0.2	84.5 ± 0.3	<.001
BMI (kg/m^2^)^a^	23.5 ± 0.8		24.2 ± 0.1	<.001		23.6 ± 0.08	24.95 ± 0.1	<.001
DM (%)	15.8 ± 1.0		17.9 ± 1.8	.33		10.7 ± 0.9	16.3 ± 1.1	<.001
HTN (%)	33.1 ± 1.4		42.9 ± 2.1	<.001		32.6 ± 1.4	53.9 ± 1.4	<.001
Smoking (%)				.25				.68
Smoker	87.3 ± 0.9		85.3 ± 1.6			8.6 ± 1.0	8.1 ± 0.9	
Past or non-smoker	12.7 ± 0.9		14.7 ± 1.6			91.4 ± 1.0	91.9 ± 0.9	
Alcohol drinking (%)				.003				<.001
None	14.0 ± 1.0		19.0 ± 1.7			22.1 ± 1.5	31.6 ± 1.7	
Mild to moderate	64.9 ± 1.4		55.3 ± 2.4			75.3 ± 1.5	65.3 ± 1.8	
Heavy	21.1 ± 1.3		62.6 ± 2.2			2.6 ± 0.6	3.1 ± 0.6	
Regular exercise (%)	20.1 ± 1.2		21.8 ± 2.1	.47		19.3 ± 1.3	19.5 ± 1.3	.91

BMI = body mass index, DM = diabetes mellitus, HTN = hypertension, OA = osteoarthritis.

* Values are expressed as mean ± standard error (SE).

### 3.2. Comparison of prevalence of radiographic knee osteoarthritis according to the frequency of beverage consumption

**Figure 2.**
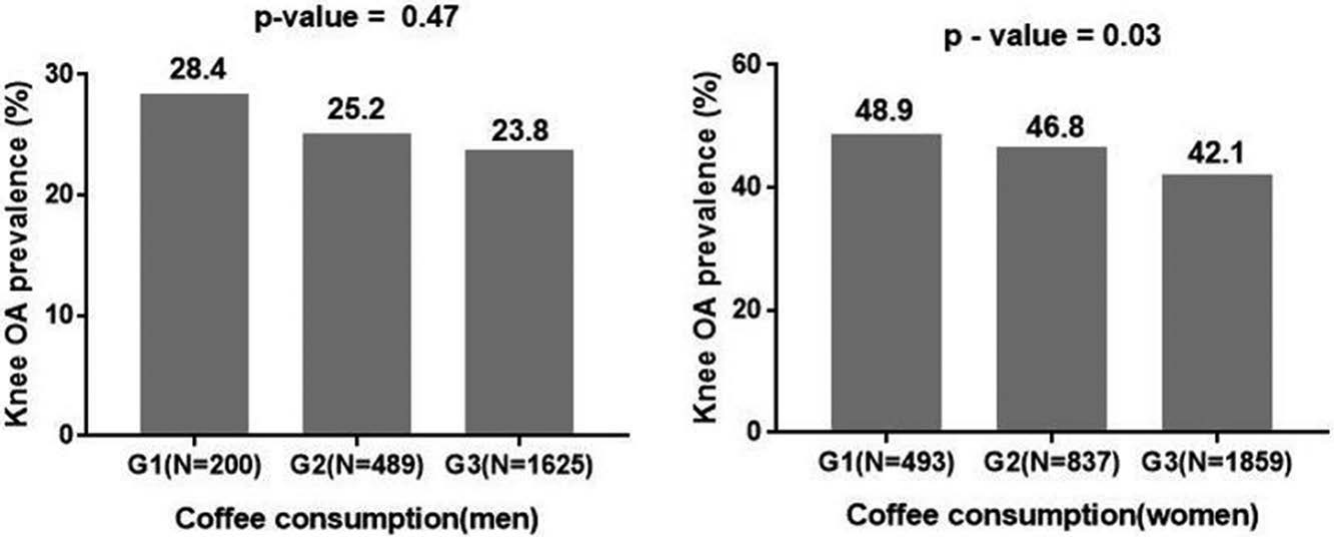
The coffee consumption and the prevalence of radiographic knee osteoarthritis. G1 = never or hardly, G2 = monthly or weekly, G3 = daily, N = number of participants, OA = osteoarthritis.

**Figure 3.**
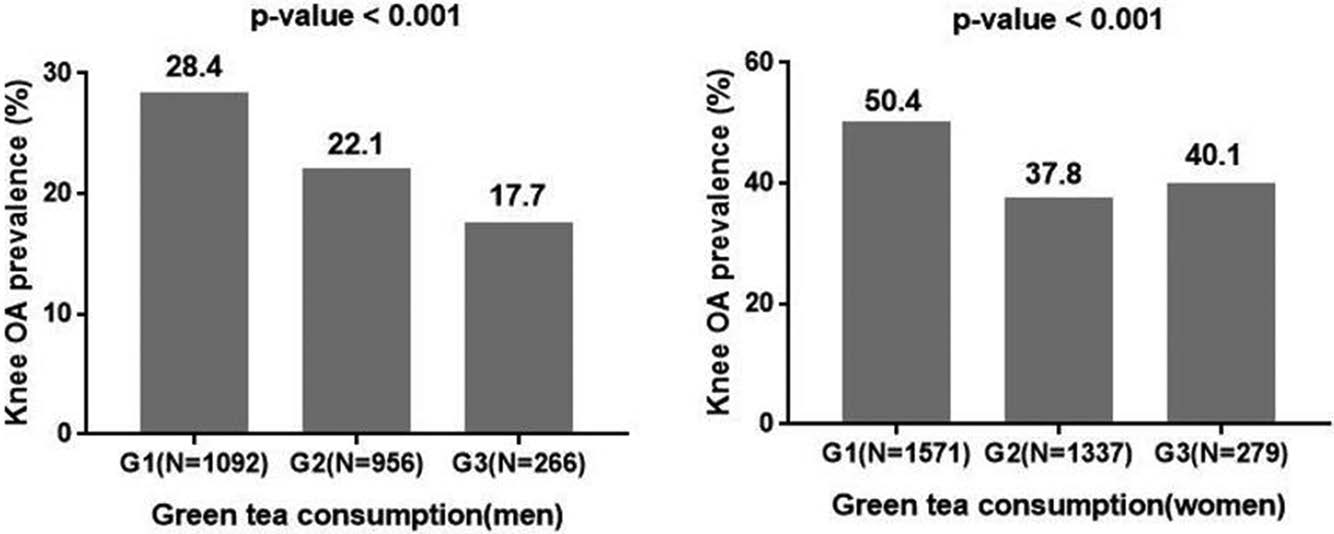
The green tea consumption and the prevalence of radiographic knee osteoarthritis. G1 = never or hardly, G2 = monthly or weekly, G3 = daily, N = number of participants, OA = osteoarthritis.

**Figure 4.**
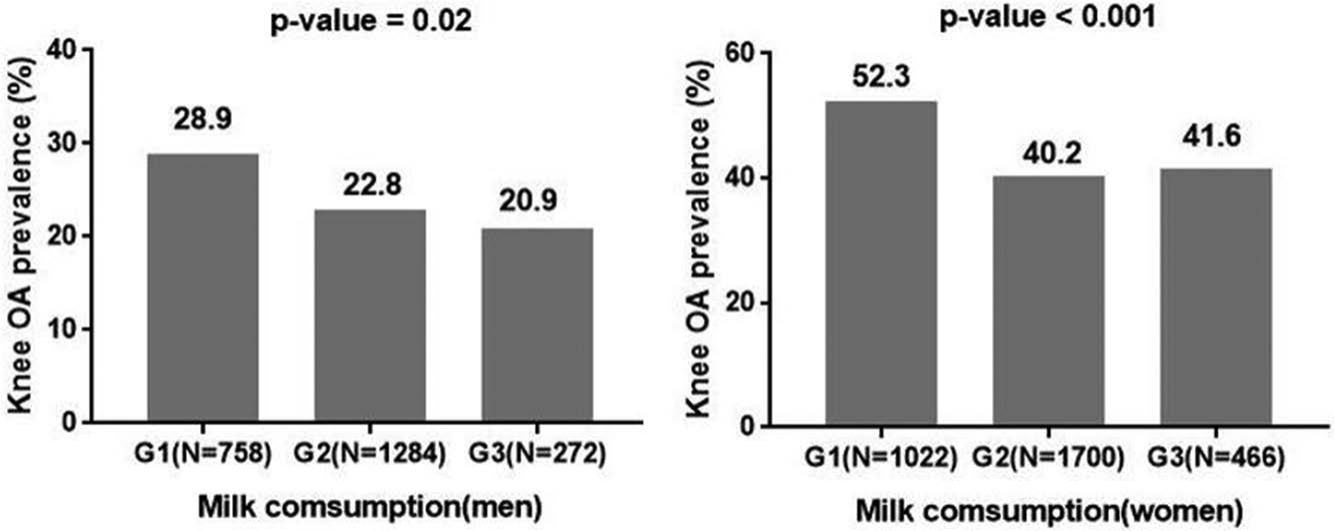
The milk consumption and the prevalence of radiographic knee osteoarthritis. G1 = never or hardly, G2 = monthly or weekly, G3 = daily, N = number of participants, OA = osteoarthritis.

**Figure 5.**
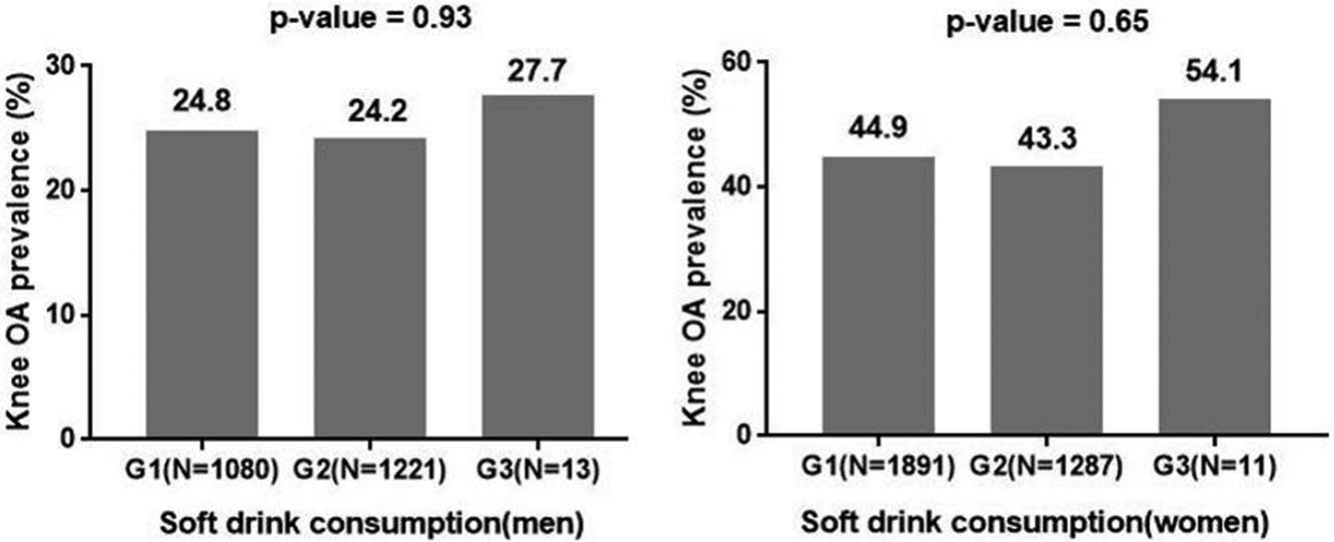
The soft drink consumption and the prevalence of radiographic knee osteoarthritis. G1 = never or hardly, G2 = monthly or weekly, G3 = daily, N = number of participants, OA = osteoarthritis.

The prevalence of radiographic knee OA tended to be negatively associated with the consumption of beverages (coffee, green tea, and milk) except soft drinks (Figs. 2–4). In addition, the consumption of coffee in men also did not show a negative relation to the prevalence of radiographic knee OA (Fig. 2). In terms of coffee, the prevalence of radiographic knee OA in men was 28.4% in G1 and 23.8% in G3 (*P* = .47), and that in women was 48.9% in G1 and 42.1% in G3 (*P* = .03, Fig. 4). However, the consumption of soft drinks was not associated with the prevalence of radiographic knee OA in men and women (*P* = .93, *P* = .65, respectively, Fig. 5).

### 3.3. The association between the frequency of beverage consumption and radiographic knee osteoarthritis after adjustment for confounding factors

The consumption of coffee in women was negatively associated with the prevalence of radiographic knee OA among the beverage after adjustment for multiple confounders. In terms of whether the participants drank each beverage, for coffee, there were significant results in women. The OR of radiographic knee OA was lower in women who have drunk at least a cup of coffee than in those who did not drink coffee (*P* for trend = .02, Table 3). However, the OR of each group did not show a significant linear trend in men (Table 3). In addition, the OR of each group did not show a significant linear trend for drinking green tea, milk, and soft drinks regardless of sex (Table 3). In terms of the frequency of beverage consumption, the OR of each group in coffee showed an increasing linear trend for radiographic knee OA (*P* for trend = .03, Table 4). Although the result was marginally significant, women who drank >1 cup of coffee daily had a lower prevalence of knee OA (OR = 0.60 95% CI = 0.41–0.89) than women who drank never or hardly (Table 4). However, the ORs of each group did not show a significant linear trend in men (*P* > .05). In addition, there was no significant linear trend of other beverages in men or women.

**Table 3:** Odds ratio for knee osteoarthritis according to whether participants were drinking or not, after adjusting for confounding factors of age, body mass index, waist circumference, alcohol drinking, smoking, diabetes mellitus, hypertension, regular exercise, and income.*

	Coffee	Green tea	Milk	Soft drink
Men				
G1	Reference	Reference	Reference	Reference
G2	1.05 (0.68–1.63)	1.13 (0.87–1.48)	1.13 (0.85–1.49)	0.81 (0.62–1.05)
*P* value	.82	.36	.40	.11
Women				
G1	Reference	Reference	Reference	Reference
G2	0.65 (0.45–0.94)	1.00 (0.78–1.26)	0.85 (0.65–1.12)	0.94 (0.72–1.22)
*P* value	.02	.97	.26	.61

G1 = never or hardly (number 0 on the self-administered questionnaire), G2 = participants who drink at least 1 cup of the beverage (number from 1 to 9 on the self-administered questionnaire).

* Values are expressed as odds ratio and 95% confidence interval.

**Table 4:** Odds ratios for knee osteoarthritis according to the frequency of beverage consumption after adjusting for confounding factors of age, body mass index, waist circumference, alcohol drinking, smoking, diabetes mellitus, hypertension, regular exercise and income.*

	Coffee	Green tea	Milk	Soft drink
Men				
G1	Reference	Reference	Reference	Reference
G2	1.05(0.64–1.71)	1.09 (0.82–1.44)	1.13 (0.85–1.50)	0.81 (0.62–1.05)
G3	1.05 (0.67–1.65)	1.34 (0.84–2.13)	1.13 (0.71–1.79)	0.48 (0.11–2.01)
*P* for trend	.97	.46	.70	.21
Women				
G1	Reference	Reference	Reference	Reference
G2	0.75 (0.50–1.12)	1.04 (0.80–1.34)	0.88 (0.66–1.16)	0.94 (0.73–1.23)
G3	0.60 (0.41–0.89)	0.82 (0.58–1.17)	0.76 (0.51–1.13)	0.42 (0.14–1.27)
*P* for trend	.03	.45	.39	.30

G1 = never or hardly (number 0 on the self-administered questionnaire), G2 = monthly or weekly (number from 1 to 6 on the self-administered questionnaire); G3, daily (number from 7 to 9 on the self-administered questionnaire).

* Values are expressed as odds ratio and 95% confidence interval.

## 4. Discussion

We found that the frequency of coffee consumption has a negative association with the prevalence of knee OA only in women. There are several unique points in our study. First, we used well-stratified data representing the general population of Korea applicable to the majority of Koreans. Second, this is the first study to consider the correlation of coffee consumption with knee OA. Lastly, our study is meaningful in that the analysis is divided by sex. To the best of our knowledge, no previous studies have taken gender into account when observing how coffee intake is correlated with the existence of radiographic knee OA.

Many studies about OA epidemiology have reported the effect of long-term coffee intake in reducing the risk of disease prognosis including total mortality, due to its anti-oxidant properties and anti-inflammatory effects.^[10,21]^ Oxidative stress plays an important role in the pathogenesis of OA,^[22]^ and recent experimental data have shown that inflammatory mediators are implicated in the OA pain process and in the degradation of the deep layer of cartilage.^[23]^ Coffee contains polyphenols including caffeic acids and chlorogenic acids, which have an antioxidant and an anti-inflammatory effect, and coffee intake increases plasma antioxidants.^[24]^ In the study by Chen et al,^[25]^ intra-articular injection of chlorogenic acid in experimental OA not only altered the expression of inflammatory mediators but also reduced cartilage degradation.

In our study, there was a significant inverse relationship between the frequency of coffee consumption and radiographic knee OA in women, but not men. Evidence suggests a protective effect of exogenous estrogen on cartilage and bone turnover.^[26]^ In postmenopausal women, the decreased level of estrogen can cause bone loss in women.^[27]^ Lower bone mineral density was associated with a higher grade of OA.^[28]^ Deletion of estrogen receptors in female mice results in cartilage damage, osteophytosis, and changes in the subchondral bone of the joints, suggesting that estrogens have a protective role in the maintenance of joint homeostasis.^[29]^ In light of the higher prevalence of OA in postmenopausal women, it can be assumed that these basic differences in hormones including estrogen may be the difference in OA development.^[30]^ Coffee can affect estrogen levels.^[31]^ Kitts presented that biologically active constituents in coffee can bind estrogen receptor protein in vitro and show weak estrogenic effects in their experimental study.^[31]^ In addition, Schliep et al^[32]^ also reported that a high caffeine intake of over 200 mg/day was associated with increased estradiol levels among Asian women. In all likelihood, these estrogenic effects of coffee would have influenced the different outcomes between men and women.

The influence of milk on OA has drawn increasing attention in recent years. Kacar et al^[13]^ reported that daily milk consumption had beneficial effects on symptomatic knee OA. Colker et al^[33]^ suggested that milk-based, bioactive micronutrient-containing drinks decreased the symptoms of OA and ameliorated the activities of daily living. A review article by Weinsier and Krumdieck^[34]^ investigated 46 studies that evaluated the relationship between milk product intake and bone health. They reported conflicting effects of dairy products including milk, yogurt, and cheese on bone health. In addition, we believe racial differences would have led to no significant result in our study. People who suffered lactose intolerance comprise about 15% of the population in northern Europeans and about 95% to 100% of American Indians and Asians.^[35]^ The other reports found that almost all adults in Singapore and Japan and 75% of adults in Korea have lactose intolerance.^[36]^ This would explain the different results of our study compared to other previous studies.

Although a few studies reported an association between green tea consumption and the risk of several disease outcomes such as metabolic syndrome and arterial stiffness, associations between green tea and knee OA have not been investigated in Asian populations.^[37]^ Green tea contains high levels of polyphenolic flavonoids, which are well known as antioxidants, and anti-inflammatory catechins.^[11]^ On the contrary, in a study by Ryu et al,^[38]^ no significant change in acute inflammatory phase reactants (C-reactive protein) and major inflammatory cytokine mediators (IL-6) was found after green tea consumption. We suggest that further research is needed on the anti-inflammatory and antioxidant effects of green tea.

In the present study, there was no significant association between soft drink consumption and radiographic knee OA. A recent study found that soda intake increased inflammatory mediators such as C-reactive protein, IL-6, and tumor necrosis factor receptor 2 concentrations.^[39]^ In addition, soda intake induces obesity and elevates inflammatory biomarkers for high glycemic load by rapidly increasing blood glucose.^[40]^ These results provide a possible explanation for the potential pathogenesis of OA due to soft drinks. Lu et al^[5]^ presented a significant inverse association between soft drink intake and joint space width of the knee in men. In their study, participants were evenly distributed among the frequency groups. However, in the present study, the percentage of participants who drank soft drinks daily was very small (men: 0.56%, women: 0.34%). We thought that this would be the reason why the association between soft drinks and knee OA was not significant in our study.

There are several limitations in the present study. First, the causal association between beverage consumption including coffee and knee OA prevalence should be interpreted with caution, because this study had a cross-sectional study design. Second, in adjusting confounding factors, other potential confounding factors such as alignment of the knee joint, possible previous knee operation like meniscectomy, and estrogen levels which affect the progression of knee OA could not be included in multivariable logistic regression analysis, because these variables were not included in KNHANES. Third, the feature of information about coffee and other beverages intake was self-reported on a short food frequency questionnaire. Therefore, non-differential classification error may have been inevitable. This classification error would attenuate the true associations. In addition, the study could not evaluate the exact amount of coffee that individuals consumed because the questionnaire in KHANES measured coffee by cups only. Fourth, we could not get information about coffee additives (sugar, cream), the method of preparation (espresso, percolated, or filtered), and the existence of caffeine; so, we could not evaluate the influences of these factors.

## 5. Conclusions

As the coffee consumption increased, the ORs of each coffee consumption frequency in relation to the radiographic knee OA group showed decreasing linear trend only in women. The ORs of other beverages consumption frequency groups did not show a significant linear trend in relation to radiographic knee OA in both sexes.

## Author contributions

**Conceptualization:** Hyung Jun Park, Jae Gyoon Kim.

**Data curation:** Bong Mo Koo, Chae Ouk Lim, Hyung Jun Park.

**Methodology:** Chae Ouk Lim, Jae Gyoon Kim.

**Project administration:** Bo Taek Kim, Jae Gyoon Kim.

**Resources:** Jae Gyoon Kim.

**Software:** Chae Ouk Lim.

**Supervision:** Jae Gyoon Kim.

**Writing – original draft:** Chae Ouk Lim.

**Writing – review & editing:** Hyung Jun Park.
